# Eighty-five percent of menu items from the six highest selling fast-food restaurants in the USA are ultra-processed

**DOI:** 10.1017/S1368980025000060

**Published:** 2025-01-30

**Authors:** Anthony J Basile, Nereus K Noshirwani, Karen L Sweazea

**Affiliations:** 1 School of Life Sciences, Arizona State University, Tempe, AZ, USA; 2 Department of Human Ecology, State University of New York at Oneonta, Oneonta, NY, USA; 3 College of Health Solutions, Arizona State University, Phoenix, AZ, USA

**Keywords:** Ultra-processed foods, Fast-food, Restaurant, Quick service, Food processing

## Abstract

**Objective:**

While fast-food is typically considered highly processed, an analysis to demonstrate this has yet to be conducted. Therefore, the objective of this research was to examine the menu items and ingredients from six fast-food restaurant menus using the NOVA classification.

**Design:**

Cross-sectional study.

**Setting:**

Data were collected from the top six highest selling US restaurants, per each food category, identified using the Quick Service and Fast Casual Restaurants (QSR) 2020 report.

**Participants:**

A total of 740 menu items were identified and classified according to their degree of processing based on ingredient lists using the NOVA classification: minimally processed (MPF), culinary processed ingredient (CPF), processed (PRF) or ultra-processed (UPF). In addition, individual ingredients that appeared on at least three menus were classified into NOVA groups, and the twenty most common ingredients were identified based on frequency of appearance in ingredient lists.

**Results:**

Across all menus, 85 % (range: 70–94 %) of items were UPF with only 11 % (range: 6–25 %) being MPF (*P* < 0·001). Additionally, 46 % of the ingredients that appeared on at least three menus were ultra-processed ingredients. Three ultra-processed ingredients appeared on all six menus: natural flavours, xanthan gum and citric acid.

**Conclusions:**

These findings show that the vast majority of menu items from major fast-food restaurants are UPF, and there are few options for MPF. Fast-food companies should consider reformulation or the addition of MPF to the menu to increase healthful food options for their patrons.

The nutritional environment plays a key role in determining eating behaviour and overall health^(1)^. Fast-food (FF) restaurants are abundant in industrialised countries as nearly a third of US adults consume FF on a daily basis^(2)^. FF and full-service restaurants account for roughly 20 % of daily calories in the USA^(3)^, while FF customers tend to underestimate how many calories they have consumed^(4)^. Over a recent 30-year span, FF restaurants have increased the variety of their menus along with the portion size, calories served and sodium content^(5)^. Greater access to FF and FF consumption have been observed as associated factors in childhood^(6,7)^ and adult^(8)^ obesity, and an increase in the density of FF restaurants is associated with an increase in BMI^(9)^. Overall, FF restaurants supply a major source of calories in the nutritional environment and thus are implicated in the current US obesity epidemic^(10)^.

Over half of the calories in the US diet come from ultra-processed foods (UPF)^(11)^, and consumption of UPF is correlated with an increased risk of various mental disorders, cardiometabolic disease and mortality outcomes^(12)^. Per the NOVA classification, UPF are defined as foods that include the fractioning of whole foods into substances, chemical modifications of these substances, assembly of unmodified and modified food substances, frequent use of cosmetic additives and sophisticated packaging^(13)^. The presence of a single ultra-processed ingredient warrants the UPF designation, and thus, it is likely that many foods in FF restaurants will be ultra-processed; however, this has not been demonstrated via a menu analysis using the NOVA classification.

Eating outside of the house is associated with increased consumption of UPF^(14)^, and among people eating at FF restaurants, it has been observed that 88 % of their calories were from UPF^(15)^, which suggests that FF restaurants serve UPF. Of note, the availability of energy-dense^(16)^ and highly palatable^(17)^ UPF in the nutritional environment has been associated with obesity^(18,19)^ and clinical trials using UPF as the independent variable have shown increased weight gain with a fully UPF diet over a 2-week period^(20)^. Therefore, both FF restaurants and UPF are implicated as contributing to the current US obesity epidemic by supplying energy-dense calories into the nutritional environment.

To determine the level of food processing of FF restaurant menus, the NOVA classification^(13)^ was used to evaluate menus from six different FF restaurants. These restaurants were those that had the highest sales within their respective restaurant category as reported by Quick Service Restaurants Magazine 2020 Top 50 Report: (Sandwich, Pizza, Burger, Snacks, Global and Chicken). There were three research questions: (1) What percentage of FF menu items are ultra-processed, and (2) what are the most common ingredients found in FF menus and what type of food processing group would the ingredient belong to? It was hypothesised that the majority of the menu items would be UPF and that some of the most common ingredients would belong to UPF.

## Methods

### Data collection

The top six highest grossing FF restaurants for each restaurant category were identified from QSR Magazine 2020 Top 50 Chart (www.qsrmagazine.com/content/qsr50-2020-top-50-chart; see online supplementary material, Supplemental Table 1). Nutritional information was gathered from each restaurant’s website (Burger: McDonald’s, Chicken: Chik-Fil-A, Global: Taco Bell, Pizza: Dominos, Sandwich: Subway and Snack: Starbucks; see online supplementary material, Supplemental Table 2). Nutritional information was presented as either purchasable menu items, individual ingredients found within food, or both (see online supplementary material, Supplemental Table 2), so the term ‘menu items’ is used to capture each of these. When ingredient information was not available for a brand-named item, the company’s website was reviewed (e.g. Starbucks sold ‘KIND® Salted Caramel & Dark Chocolate Nut Bar’ and the ingredient information was collected from www.kindsnacks.com). Alcoholic beverages, items from a specific version of restaurants (e.g. Cantina menu for Taco Bell) and carry-home items (e.g. the ‘at-home items’ from Starbucks) were not included in the analysis. In total, 872 menu items were collected across restaurants, and after removing duplicate items and items without ingredient information available (see online supplementary material, Supplemental Table 2), the remaining 748 items were then included in the analysis.

### Analysis of menu items

To answer the research questions, the proportion of menu items for each NOVA group was determined with a mean calculated for all restaurants. Based on ingredient information, menu items were coded into the four NOVA classification groups: minimally processed (MPF), culinary processed (CPF), processed (PRF) and UPF^(11)^. Coding was performed independently to agreement by NKN and AJB. Coders initially agreed on 93 % of all menu items (Chicken: 93 %, Pizza: 85 %, Burger: 96 %, Snack: 92 %, Sandwich: 93 % and Global: 100 %). Data were presented in a 100 % stacked column using Microsoft Excel. SPSS was used to conduct non-parametric single *χ*^2^ tests to determine if there was a difference in the proportion of menu items in the processing groups for each menu and for all menu items (SPSS 29; IBM).

### Ingredient analysis

Using Monkeylearn.com, word/phrase frequency clouds were created to visualise the top fifty words/phrases within and across menu items, where the larger the word/phrase appears, the more frequently it appeared in the ingredient list. From there, the top ingredients in each menu (words/phrases identified from monkeylearn.com) were ranked (i.e. the most and least common appearing ingredient were ranked from 1 to 50, respectively). Ingredient lists were then merged, the number of times an ingredient was listed was identified, and the mean rank and sd were calculated. Ingredients that appeared in half or more of the food menus were presented, and functional classes were identified from Codex General Standard for Food Additives (GSFA) from the FAO of the UN and WHO^(21)^. Ingredients were then coded independently and to agreement by NKN and AJB (77 % initial agreement) into five groups: MPF, CPF, PRF, UPF and NTR (vitamins, minerals and water).

## Results

### Food processing percentage

**Figure 1.**
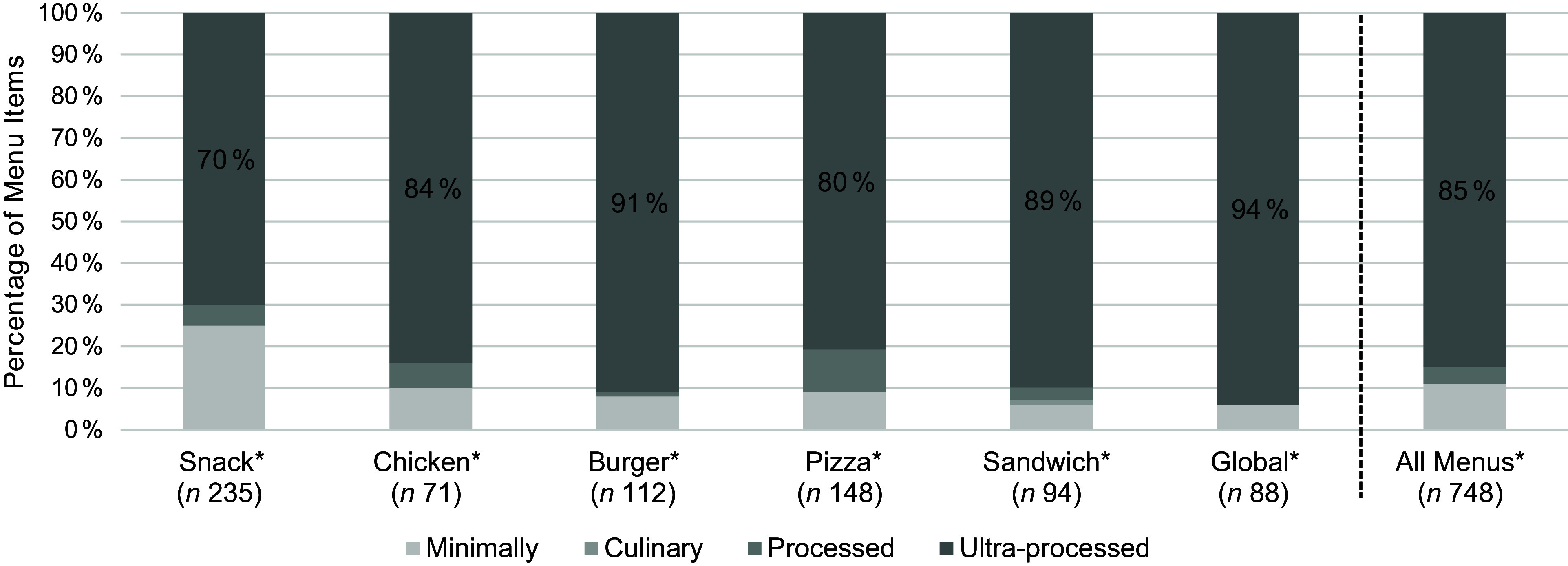
Proportion of menu items from each food processing group for six fast-food restaurant menus. *P < 0·001 for all; single sample *χ*^2^ test.

Figure 1 shows the percentage of menu items for each NOVA classification group for each restaurant menu and all menus. The majority of menu items in each restaurant (range: 70–94 %), and across restaurants (85 %), were ultra-processed (*P* < 0·001 for all menus and for all menu items across menus). In addition, MPF comprised only 11 % of items on average across menus.

### Ingredient analysis

**Figure 2.**
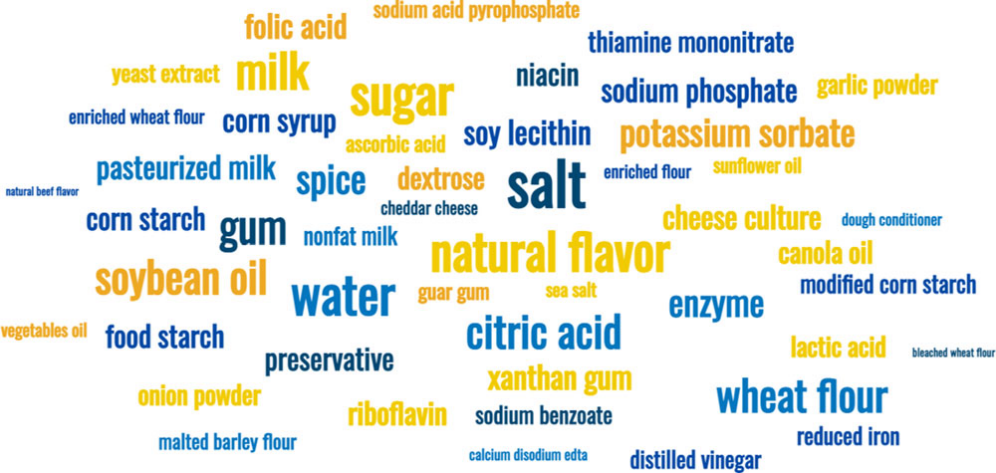
Ingredient word cloud from six fast-food restaurant menu items. Word/phrase frequency cloud produced from the top fifty words from all menu items.

Figure 2 depicts the top fifty ingredients across the six chosen restaurants, where the larger the word appears the more frequently it appears in the menus. Thirty-nine ingredients appeared in half or more of the menus (Table 1). Salt and sugar, both CPF, were the two highest-ranked ingredients. Only three UPF ingredients appeared in all six menus: natural flavour, citric acid and xanthan gum (listed in descending mean rank). Of the thirty-nine ingredients, 15 % were CPF, 18 % were NTR, 21 % were MPF and 46 % were UPF, with zero PRF ingredients. Fifteen of the ingredients were listed in the Codex General Standard for Food Additives, and most (80 %) were UPF ingredients. The top three common UPF functional classes were emulsifier (*n* 8 appearances), thickener (*n* 6), and sequestrant and stabiliser (*n* 5).

**Table 1:** Ingredients appearing in three or more of analysed fast-food restaurant menus

Ingredient	Menu count	Mean rank	sd	NOVA group	Functional class
Salt	6	1·17·	0·41	CPF	n/a
Sugar	6	3·17·	1·60	CPF	n/a
Natural flavour	6	4·33·	1·03	UPF	n/a
Citric acid	6	9·17·	3·87	UPF	Acidity regulator, antioxidant, color retention agent, sequestrant
Soyabean oil	6	10·50·	8·73	CPF	n/a
Xanthan gum	6	25·17·	11·20	UPF	Emulsifier, foaming agent, stabiliser, thickener
Water	5	2·40·	0·55	NTR	n/a
Wheat flour	5	9·20·	6·14	MPF	n/a
Folic acid	5	16·20·	9·47	NTR	n/a
Potassium sorbate	5	16·20·	7·19	UPF	Preservative
Soya lecithin	5	17·80·	5·89	UPF	Antioxidant, emulsifier
Cheese culture	5	21·60·	10·06	UPF	n/a
Thiamine mononitrate	5	23·40·	6·88	NTR	n/a
Spice	4	5·75·	2·06	MPF	n/a
Riboflavin	4	13·00·	2·45	NTR	Colour
Niacin	4	14·00·	2·45	NTR	n/a
Sodium phosphate	4	18·25·	9·22	UPF	Acidity regulator, emulsifier, emulsifying salt, humectant, raising agent, sequestrant, stabiliser, thickener.
Canola oil	4	22·00·	5·48	CPF	n/a
Modified corn starch	4	25·00·	8·45	UPF	Emulsifier, thickener*
Reduced iron	4	25·75·	7·41	NTR	Colour (listed as iron oxides)
Sodium benzoate	4	27·75·	8·96	UPF	Preservative
Malted barley flour	4	29·25·	9·91	MPF	n/a
Modified food starch	4	29·25·	17·76	UPF	Emulsifier, thickener*
Sodium acid pyrophosphate	4	36·75·	12·89	UPF	n/a
Calcium disodium EDTA	4	44·25·	3·20	UPF	Colour retention agent, preservative, sequestrant
Enzyme	3	6·67·	2·52	UPF	n/a
Dextrose	3	11·33·	2·08	UPF	Emulsifier, stabiliser, thickener (listed as dextrins, roasted starch)
Garlic powder	3	17·67·	3·06	MPF	n/a
Onion powder	3	19·67·	8·08	MPF	n/a
Pasteurised milk	3	20·67·	11·02	MPF	n/a
Lactic acid	3	21·00·	12·29	UPF	Acidity regulator, emulsifier, sequestrant, stabiliser
Distilled vinegar	3	21·67·	3·51	CPF	n/a
Yeast extract	3	22·00·	16·64	UPF	n/a
Corn syrup	3	22·67·	16·50	UPF	n/a
Non-fat milk	3	23·33·	14·47	MPF	n/a
Guar gum	3	28·33·	10·21	UPF	Emulsifier, stabiliser, thickener
Enriched wheat flour	3	32·33·	12·01	MPF	n/a
Ascorbic acid	3	32·67·	9·61	NTR	Acidity regulator, antioxidant, flour treatment agent, sequestrant
Sunflower oil	3	36·33·	8·14	CPF	n/a

CPF, culinary processed; UPF, ultra-processed foods; NTR, nutrient and water; MPF, minimally processed.

Data developed from the top fifty appearing words/phrases in each menu; functional class determined via the Codex General Standard for Food Additives (GSFA) from the FAO of the UN and WHO.

## Discussion

This was the first study to explore the level of food processing among menus for several popular FF restaurants in the USA using the NOVA classification. The results show that, on average, FF restaurant menu items are highly processed with 85 % of menu items being ultra-processed foods and only 11 % of menu items consisting of minimally processed foods. Thus, there are very few non-UPF options available at these six FF restaurants. Given the role of the food environment in health, FF restaurants may be contributing to the rise in obesity in the USA by providing predominantly energy-dense, ultra-processed foods to their customers. In addition, across ingredients that appeared in three or more of the menus, 46 % were considered ultra-processed, with the most common functional classes being emulsifier, thickener, and sequestrant and stabiliser. The three most frequently appearing ultra-processed ingredients in the FF restaurant menu items were natural flavours, citric acid and xanthan gum.

Considering that nearly a third of US adults consume FF on a daily basis^(2)^, and the association between FF restaurant proximity, FF consumption and UPF consumption with obesity^(6–10,18,19)^, these results are of concern for public health. This prevalence of UPF across these six FF restaurants is higher than what has been observed in grocery stores, where the majority of items are also UPF^(16)^. Together, between the abundance of UPF at grocery stores and FF restaurants, consumers may need to go out of their way to seek non-UPF. Future research examining the prevalence of UPF in non-FF restaurants is warranted to better describe additional sources of food within the US nutritional environment. Public health efforts to decrease consumption of UPF and increase consumption of MPF, at every level of the nutritional environment, are warranted to combat the obesity epidemic.

Natural flavours were the most common UPF ingredient, which appeared in every FF restaurant that was analysed. According to the US Food and Drug Administration (FDA), natural flavours are anything (e.g. essential oil, extract and protein) collected from foods (e.g. spice, fruit, vegetables, herbs, etc.) whose function is flavouring, rather than nutritional^(22)^. While there is debate whether natural flavours are ‘natural’^(23)^, the presence of them within a food item warrants the classification of an UPF. The next two most popular UPF ingredients were citric acid and xanthan gum. Citric acid (an organic acid) is currently the single largest chemical obtained from chemical biosynthesis, and its popularity as a food additive is due to its chemical nature (multiple functions within food)^(24)^. In an analysis of ingredients used in culinary preparations from institutional food services (e.g. private cafeterias and universities), 8·4–12·6 % of ingredients were UPF and were mainly used in protein dishes and desserts^(25)^. This further demonstrates the invasiveness of ultra-processed ingredients used in food preparation.

A critique of the NOVA classification is the inclusion of specific ingredients as a method to identify UPF because, while citric acid is a food additive, citric acid is also found naturally in foods^(26)^. Xanthan gum is a naturally occurring microbial exopolysaccharide, and it is considered safe by the FDA but is not digestible by humans^(27)^. Emulsifiers form a uniform texture consistency and are abundant in UPF. This, there is concern about the impact of emulsifiers on the pathogenesis of certain diseases^(28)^. While natural flavours, citric acid and xanthan gum are all ambiguous in their effects on human health, collectively, these three ingredients make UPF shelf stable and more flavourful, which may increase palatability and promote increased consumption.

This analysis has some strengths and limitations. First, the sample size was limited to six restaurants; however, these six represented the highest grossing restaurant for each restaurant category and thus represented the most frequented FF restaurants. In addition, selecting one from each of the restaurant types allowed for a broad menu analysis capturing the variety of FF options available within the USA. However, because we only chose one restaurant per food category, our findings cannot be applied to other FF restaurants. Another limitation is that some menu items were removed from analysis because the restaurants or Internet did not provide ingredient information; thus, the menu and ingredient analyses were produced with only available data. While the NOVA classification is one of the most common food processing classifications, the definition of ultra-processed foods has changed considerably over time^(29)^; thus, as new food processing definitions and categorisations are developed, the results of this study may differ.

This study illustrates the invasive nature of ultra-processed foods in the US nutritional environment. The high intake UPF in the US could also be due to the low availability of healthful, nutrient-dense, minimally processed food choices among some of the highest-grossing FF restaurants in the USA. Therefore, to improve the nutritional quality of their menu items, FF companies should consider reformulation or the addition of MPF to the menu to increase healthful food options.

## Supporting information

Basile et al. supplementary material 1 Basile et al. supplementary material

Basile et al. supplementary material 2 Basile et al. supplementary material

## Supplementary material

For supplementary material accompanying this paper visit https://doi.org/10.1017/S1368980025000060

## References

[1] Glanz K , Sallis JF , Saelens BE et al. (2005) Healthy nutrition environments: concepts and measures. Am J Heal Promot 19, 330–333. 10.4278/0890-1171-19.5.330 15895534

[2] Fryar CD , Hughes JP , Herrick KA et al. (2018) Fast food consumption among adults in the United States, 2013–2016. NCHS Data Brief 322, 1–8. 30312154

[3] Mazidi M & Speakman JR (2017) Higher densities of fast-food and full-service restaurants are not associated with obesity prevalence. Am J Clin Nutr 106, 603–613. 28566310 10.3945/ajcn.116.151407

[4] Block JP , Condon SK , Kleinman K et al. (2013) Consumers’ estimation of calorie content at fast food restaurants: cross sectional observational study. BMJ 346, f2907–f2907. 23704170 10.1136/bmj.f2907 PMC3662831

[5] McCrory MA , Harbaugh AG , Appeadu S et al. (2019) Fast-food offerings in the United States in 1986, 1991, and 2016 show large increases in food variety, portion size, dietary energy, and selected micronutrients. J Acad Nutr Diet 119, 923–933. 30826304 10.1016/j.jand.2018.12.004

[6] Jia P , Shi Y , Jiang Q et al. (2023) Environmental determinants of childhood obesity: a meta-analysis. Lancet Glob Heal 11, S7. 10.1016/S2214-109X(23)00092-X 36866484

[7] Jakobsen DD , Brader L & Bruun JM (2023) Association between food, beverages and overweight/obesity in children and adolescents-a systematic review and meta-analysis of observational studies. Nutrients 15, 764. 36771470 10.3390/nu15030764 PMC9920526

[8] Reitzel LR , Regan SD , Nguyen N et al. (2014) Density and proximity of fast food restaurants and body mass index among African Americans. Am J Public Health 104, 110–116. 23678913 10.2105/AJPH.2012.301140 PMC3910025

[9] Acciai F , DeWeese RS , Yedidia MJ et al. (2022) Differential associations between changes in food environment and changes in BMI among adults living in urban, low-income communities. J Nutr 152, 2582–2590. 36774124 10.1093/jn/nxac186 PMC9644168

[10] Temple NJ (2022) The origins of the obesity epidemic in the USA-lessons for today. Nutrients 14, 4253. 36296935 10.3390/nu14204253 PMC9611578

[11] Marino M , Puppo F , Del Bo’ C et al. (2021) A systematic review of worldwide consumption of ultra-processed foods: findings and criticisms. Nutrients 13, 2778. 34444936 10.3390/nu13082778 PMC8398521

[12] Lane MM , Gamage E , Du S et al. (2024) Ultra-processed food exposure and adverse health outcomes: umbrella review of epidemiological meta-analyses. BMJ 384, e077310. 38418082 10.1136/bmj-2023-077310 PMC10899807

[13] Monteiro CA , Cannon G , Levy RB et al. (2019) Ultra-processed foods: what they are and how to identify them. Public Health Nutr 22, 936–941. 30744710 10.1017/S1368980018003762 PMC10260459

[14] Andrade GC , Gombi-Vaca MF , da Louzada MLC et al. (2020) The consumption of ultra-processed foods according to eating out occasions. Public Health Nutr 23, 1041–1048. 31544732 10.1017/S1368980019002623 PMC10200589

[15] Souza TN , Andrade GC , Rauber F et al. (2022) Consumption of ultra-processed foods and the eating location: can they be associated? Br J Nutr 128, 1587–1594. 34915943 10.1017/S0007114521004992

[16] Gupta S , Hawk T , Aggarwal A et al. (2019) Characterizing ultra-processed foods by energy density, nutrient density, and cost. Front Nutr 6, 1–9. 31231655 10.3389/fnut.2019.00070 PMC6558394

[17] Fardet A (2016) Minimally processed foods are more satiating and less hyperglycemic than ultra-processed foods: a preliminary study with 98 ready-to-eat foods. Food Funct 7, 2338–2346. 27125637 10.1039/c6fo00107f

[18] de Araújo TP , de Moraes MM , Magalhães V et al. (2021) Ultra-processed food availability and noncommunicable diseases: a systematic review. Int J Environ Res Public Health 18, 7382. 34299832 10.3390/ijerph18147382 PMC8306957

[19] Vitale M , Costabile G , Testa R et al. (2024) Ultra-processed foods and human health: a systematic review and meta-analysis of prospective cohort studies. Adv Nutr 15, 100121. 38245358 10.1016/j.advnut.2023.09.009 PMC10831891

[20] Hall KD , Ayuketah A , Brychta R et al. (2019) Clinical and translational report ultra-processed diets cause excess calorie intake and weight gain: an inpatient randomized controlled trial of ad libitum food intake cell metabolism clinical and translational report ultra-processed diets cause excess Ca. Cell Metab 30, 1–11.31204280

[21] Food and Agriculture Organization (2015) Codex General Standard for Food Additives. Rome: Food and Agriculture Organization.

[22] U.S. Food and Drug Administration (2024) Code of Federal Regulations Title 21. Silver Spring, MD: United States Food and Drug Administration.

[23] Goodman MJ (2017) The “natural” vs. “natural flavors” conflict in food labeling: a regulatory viewpoint. Food Drug Law J 72, 78–102. 29140655

[24] Ciriminna R , Meneguzzo F , Delisi R et al. (2017) Citric acid: emerging applications of key biotechnology industrial product. Chem Cent J 11, 22. 28326128 10.1186/s13065-017-0251-y PMC5342991

[25] Padovan M , Thimoteo da Cunha D , Adriano Martins C et al. (2023) Ultra-processed foods in institutional food services: what are diners eating? Arch Latinoam Nutr 73, 8–18.

[26] Gibney MJ & Forde CG (2022) Nutrition research challenges for processed food and health. Nat Food 3, 104–109. 37117956 10.1038/s43016-021-00457-9

[27] Abu Elella MH , Goda ES , Gab-Allah MA et al. (2021) Xanthan gum-derived materials for applications in environment and eco-friendly materials: a review. J Environ Chem Eng 9, 104702.

[28] Sandall A , Smith L , Svensen E et al. (2023) Emulsifiers in ultra-processed foods in the UK food supply. Public Health Nutr 26, 2256–2270. 37732384 10.1017/S1368980023002021 PMC10641632

[29] Gibney MJ (2019) Ultra-processed foods: definitions and policy issues. Curr Dev Nutr 3, nzy077. 30820487 10.1093/cdn/nzy077 PMC6389637

