# Supplementary figures and images for: Eighty-five percent of menu items from the six highest selling fast-food restaurants in the USA are ultra-processed
Source: Public Health Nutr. 2025 Jan 30;28(1):e53. doi: 10.1017/S1368980025000060 (PMC11983999; doi:10.1017/S1368980025000060)

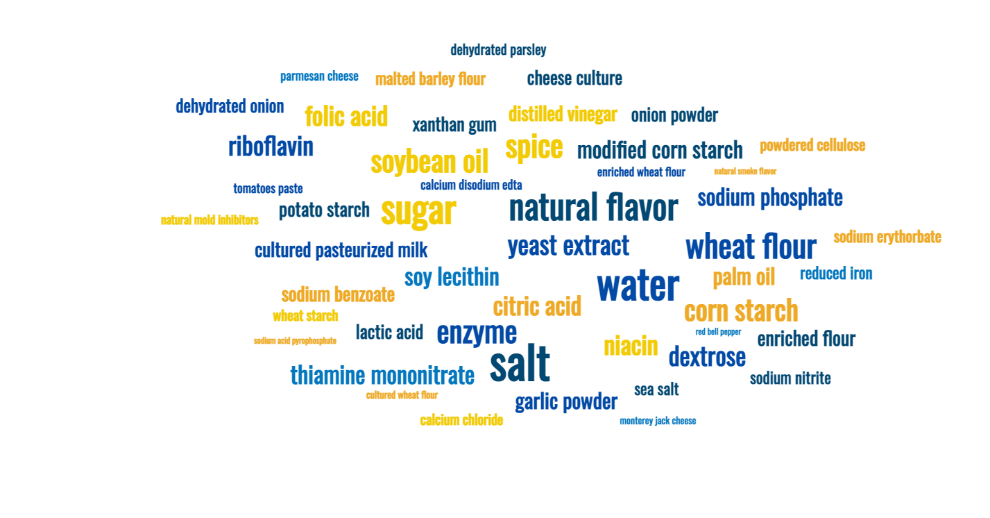

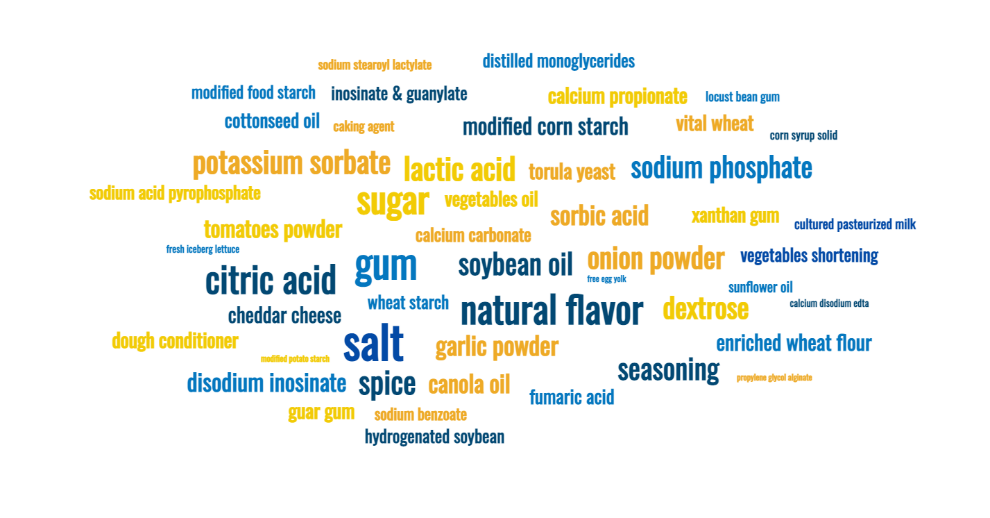

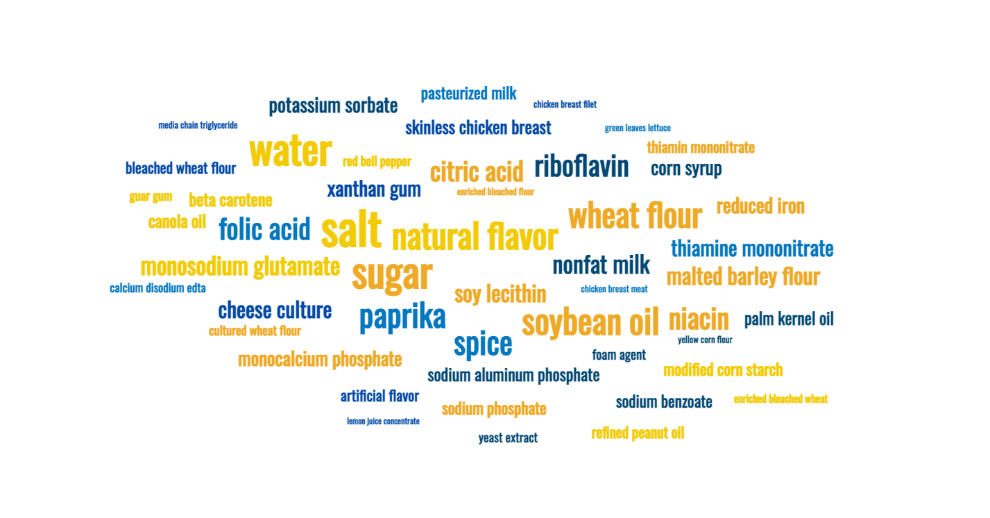

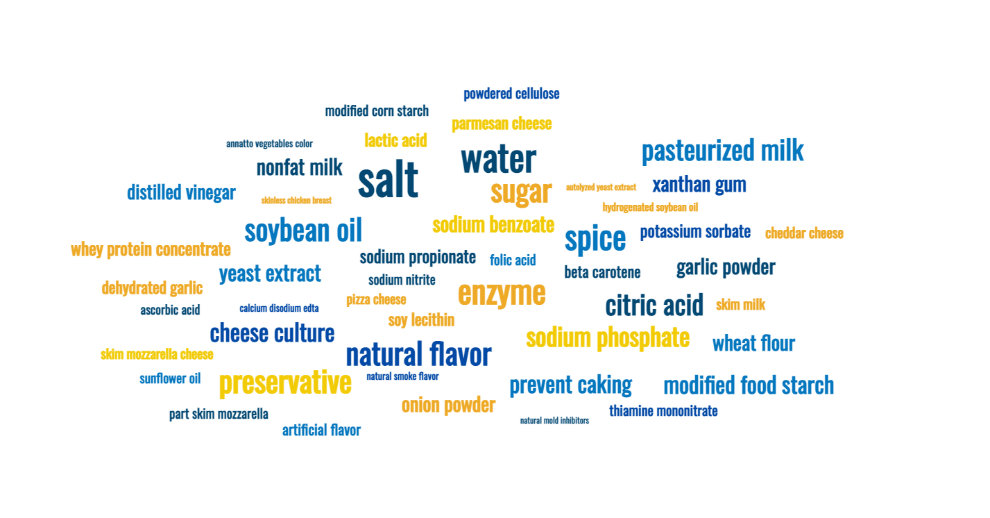

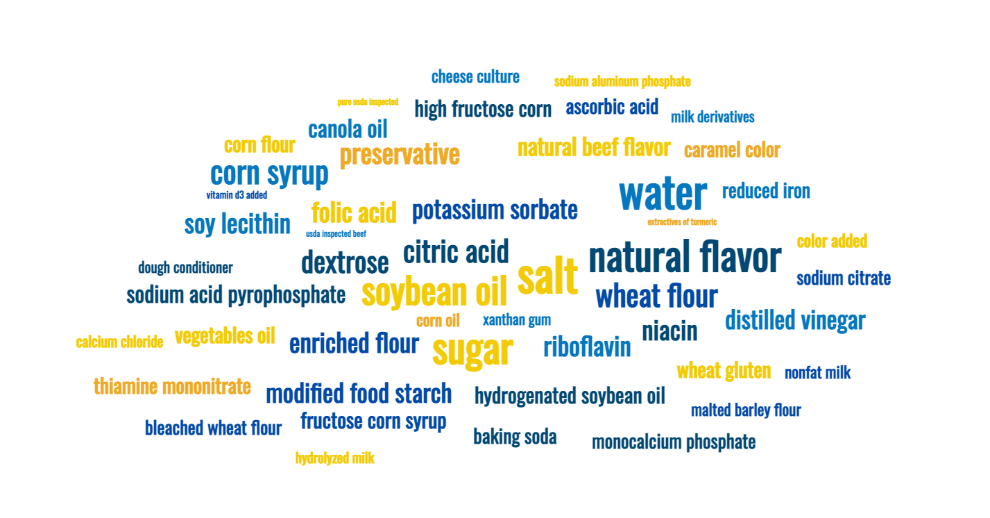

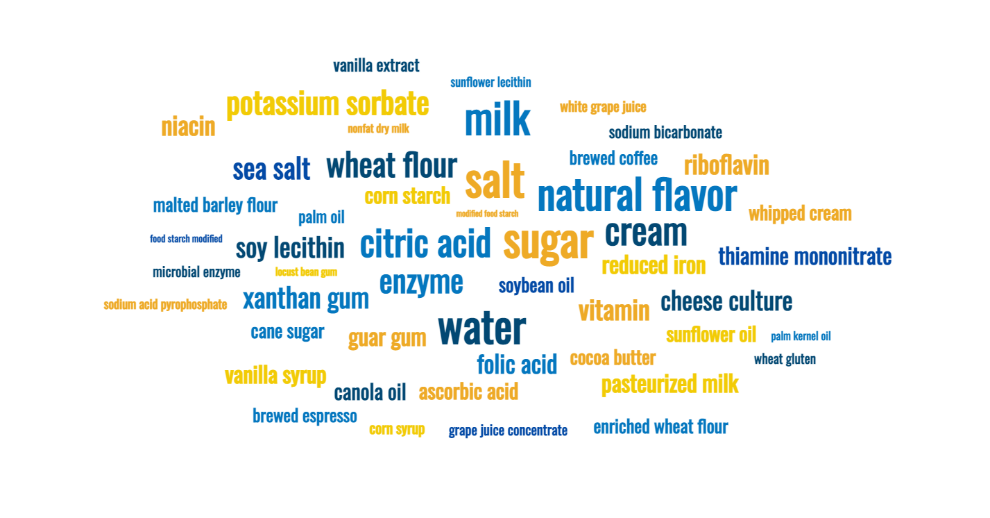


**Sandwich:**

**Global:**

**Chicken:**

**Pizza:**

**Burger:**

**Snack:**

Supplement: Basile et al. supplementary material 1 — Basile et al. supplementary material [file S1368980025000060sup001.docx]
