# Supplementary material for: Eighty-five percent of menu items from the six highest selling fast-food restaurants in the USA are ultra-processed
Source: Public Health Nutr. 2025 Jan 30;28(1):e53. doi: 10.1017/S1368980025000060 (PMC11983999; doi:10.1017/S1368980025000060)
Supplement: Basile et al. supplementary material 2 — Basile et al. supplementary material [file S1368980025000060sup002.docx]

Supplemental Table 1: Fast-Food Restaurant Analytics

| **Overall Ranking** | **Company Name** | **Restaurant Category** | **2019 US Systemwide Sales (Millions)** | **2019 Total Units** |
| --- | --- | --- | --- | --- |
| 1 | McDonald's | Burger | 40,413 | 13,846 |
| 2 | Starbucks | Snack | 21,550 | 15,041 |
| 4 | Taco Bell | Global | 11,000 | 7,089 |
| 3 | Chick-Fil-A | Chicken | 11,000 | 2,500 |
| 6 | Subway | Sandwich | 10,000 | 23,802 |
| 9 | Domino's | Pizza | 7,100 | 6,157 |
| Note: Data from https://www.qsrmagazine.com/content/qsr50-2020-top-50-chart | | | | |

Supplemental Table 2: Menu Data Collection Process

| **Restaurant** | **Date Collected** | **Menu Type** | **Original Items** | **Duplicates**  **Merged** | **Unanalyzed Items*** | **Analyzed**  **Items** |
| --- | --- | --- | --- | --- | --- | --- |
| McDonald's | 3/13/2021 | Menu items | 112 | 0 | 0 | 112 |
| Starbucks | 3/14/2021 & 3/17/2021 | Menu items | 313 | 3 🡪 1 | 76 | 235 |
| Taco Bell | 1/19/2021 | Menu items | 120 | 33 🡪 22 | 0 | 88 |
| Chick-Fil-A | 3/15/2021 | Menu items | 85 | 2 🡪 1 | 13 | 71 |
| Subway | 1/19/2021 | Ingredients | 94 | 0 | 0 | 94 |
| Domino's | 2/3/2021 | Menu items & Ingredients | 148 | 0 | 0 | 148 |
| Note: *Items without ingredient information available | | | | | | |
